# Prognostic Implication of Intestinal Wall Edema in Patients with Aortic Stenosis Receiving Trans-Catheter Aortic Valve Replacement

**DOI:** 10.3390/jcm12247658

**Published:** 2023-12-13

**Authors:** Kousuke Akao, Teruhiko Imamura, Shuhei Tanaka, Hiroshi Onoda, Ryuichi Ushijima, Mitsuo Sobajima, Nobuyuki Fukuda, Hiroshi Ueno, Koichiro Kinugawa

**Affiliations:** The Second Department of Internal Medicine, University of Toyama, Toyama 930-0194, Japan; apollon080607@gmail.com (K.A.); hueno@med.u-toyama.ac.jp (H.U.);

**Keywords:** heart failure, hemodynamics, congestion, valvular disease, aortic valve disease

## Abstract

Background: A recently proposed mechanism, the intestinal–cardiovascular relationship, serves as a framework to elucidate the interplay between these two systems. In our investigation, we assessed the prognostic implications of colon wall thickness, a marker correlated with intestinal congestion and dysfunction, in patients diagnosed with severe aortic stenosis undergoing transcatheter aortic valve replacement (TAVR). Methods: Patients diagnosed with severe aortic stenosis who underwent TAVR at our institution during the period spanning 2015 to 2022 were retrospectively enrolled. As part of the institutional protocol, patients underwent abdominal computed tomography upon admission, preceding TAVR. Our analysis aimed to assess the influence of colon wall thickness on the occurrence of either all-cause mortality or readmission due to heart failure within a two-year period. Results: A total of 345 patients were included. The median age was 85 (82, 88) years, and 99 patients were male. Baseline colon wall thickness was distributed widely, with a median value of 2.2 (2.0, 2.5) mm. Patients with thicker colon walls tended to have lower pulmonary artery pulsatility index values, indicating more impaired right ventricular function and more advanced malnutrition. A thicker colon wall was independently associated with 2-year death or heart failure readmission with a hazard ratio of 2.02 (95% confidence interval 1.01–14.07), adjusted for hemoglobin, age, and plasma B-type natriuretic peptide levels (*p* = 0.049), and significantly stratified the primary endpoint at a cutoff of 2.7 mm (25% versus 10%, *p* = 0.005). Conclusions: Our initial observation revealed that a thicker baseline colon wall correlated with increased rates of mid-term mortality and readmission due to heart failure subsequent to TAVR. Developing a comprehensive understanding of the underlying causality necessitates further in-depth investigations through subsequent studies.

## 1. Background

Trans-catheter aortic valve replacement (TAVR) is a less invasive trans-catheter intervention for severe aortic stenosis and is indicated for low- to high-surgical-risk patients [1,2,3]. However, post-procedural mid-term mortality and morbidity remain high [4]. Risk stratification and peri-procedural management are expected to be improved further.

TAVR can ameliorate stenotic aortic valve, whereas residual extra-valvular and extra-cardiac impairments may persist even after TAVR, including left ventricular dysfunction, left atrial remodeling, pulmonary hypertension, right ventricular failure, other valvular diseases, end-organ dysfunction, and systemic cachexia [5]. As most TAVR candidates are elderly with multiple comorbidities, these impairments may be the next target to further improve patients’ clinical outcomes after TAVR [6].

Recently, the interaction between the intestine and the cardiovascular system, which is called intestinal–cardiovascular syndrome, has been increasingly recognized as playing a pivotal role in the progression of cardiovascular diseases [7,8]. Heart failure triggers intestinal dysfunction, which can be assessed by the thickness of the colon wall [9], via impaired intestinal blood flow and congestion [10,11]. The progression of intestinal dysfunction stimulates dysbiosis and systemic inflammation, which further advances heart failure [12]. Intestinal dysfunction causes constipation, which increases afterload on the left ventricle [13]. The presence of intestinal dysfunction decreases the absorption of nutrients and progresses to malnutrition and sarcopenia [9].

Some studies showed the association between cardiac function and morphologic features of the gut in the heart failure cohort [14]. However, no studies evaluated the clinical implication of intestinal dysfunction in patients with severe aortic stenosis receiving TAVR. Given that most of these patients have cardiac diastolic dysfunction due to incremental afterload on the left ventricle, we hypothesized that the presence of intestinal wall edema may have an association with several heart failure-related clinical parameters and also have a negative prognostic impact via intestinal–cardiovascular syndrome. 

In this study, we aimed to clarify the prognostic impact of colon wall thickness (CWT) in patients with severe aortic stenosis receiving TAVR. 

## 2. Methods

### 2.1. Patient Selection

Consecutive patients with severe aortic stenosis who underwent TAVR at a single academic large center between 2015 and 2022 were prospectively enrolled in our institutional registry database and were included retrospectively in this study. Patients underwent abdominal computed tomography on admission prior to TAVR as an institutional protocol. CWT was measured as detailed below using the obtained computed tomography imaging. Patients with missing data were excluded. Patients with a history of gastrointestinal disease were excluded, including gastrointestinal cancer, enteritis, infectious colitis, ischemic colitis, inflammatory bowel disease, and ileus. Written informed consent was obtained from all participants on admission. The institutional review board approved the study protocol. 

### 2.2. Measurement of CWT

**Figure 1:**
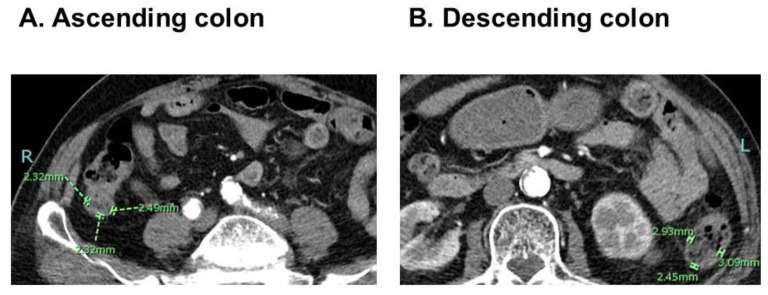
Measurements of CWT in the abdominal computed tomography. CWT was measured 3 times at the different positions of ascending colon (**A**) and descending colon (**B**), respectively. A total of 6 CWTs were obtained and averaged. In this representative case, the averaged CWT was calculated as 2.6 mm.

**Figure 2:**
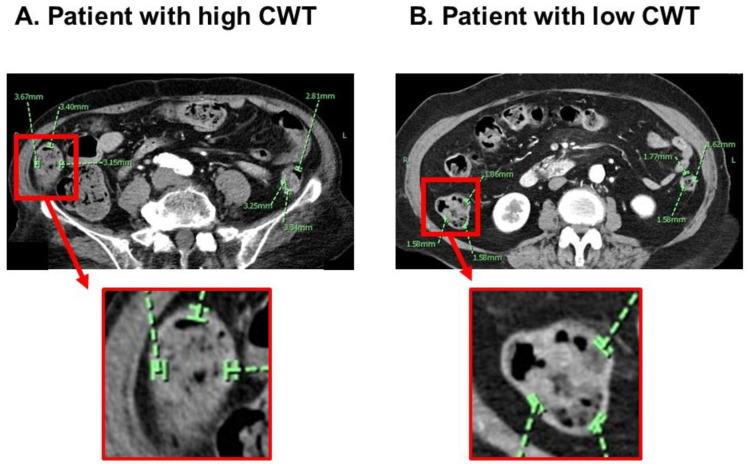
Representative of a patient with high CWT (**A**) and another with low CWT (**B**).

On admission to the index hospitalization before TAVR, all patients underwent abdominal computed tomography imaging utilizing a third-generation 192-slice dual-source computed tomography scanner (Somatom Force, Siemens Healthcare, Forchheim, Germany). The scans were conducted at a tube voltage of either 70 kV or 120/100 kV, employing a gantry rotation time of 0.25 s and a detector collimation of 2 × 192 × 0.6 mm. All images were retrospectively analyzed by the researcher (AK) who was blind to the data of this study at the time of the measurements. Another researcher (TI) also measured CWT to assess the inter-observer reliability of this methodology. CWT was measured at the inner luminal and outer mesenteric aspects on trans-axial projections of the bowel in the area of a well-distended colon whenever possible on the basis of the previous literature [14,15]. The average CWT was calculated from the averages of each of the three points among the ascending colon and descending colon (Figure 1A,B). Representatives of a patient with high CWT and another with low CWT were displayed in Figure 2A,B.

### 2.3. Other Baseline Characteristics

Baseline demographics, laboratory, echocardiographic, and medication data collected during the index hospitalization prior to TAVR were gathered alongside CWT data as foundational characteristics. 

### 2.4. TAVR Procedure

Patients with symptomatic severe aortic stenosis with a peak velocity of >4.0 m/s, a mean pressure gradient of >40 mm Hg, or an aortic valve area of <1.0 cm^2^ were eligible for TAVR. The indication for TAVR was eventually determined by the heart valve team conference, which consisted of cardiac surgeons, interventional cardiologists, anesthesiologists, nurses, and imaging specialists. 

Patients underwent the standard TAVR procedure using the Edwards Sapien XT/Sapien 3 Transcatheter Heart Valve (Edwards Lifesciences, Irvine, CA, USA) or the Medtronic CoreValve/Evolut R Revolving System (Medtronic, Minneapolis, MN, USA). An antithrombotic regimen following TAVR was used at the discretion of the attending physician. 

### 2.5. Post-TAVR Course and Primary Outcome

Patients were followed at our center or affiliated centers by board-certified cardiologists every 1–2 months in a standard manner. Medications were adjusted at the discretion of the attending physicians. Day 0 was defined as the time of the TAVR procedure. The observation period was 2 years or until May 2023 from day 0. The primary outcome was a composite of all-cause death and heart failure readmissions. 

### 2.6. Statistical Analysis

Continuous variables were presented as median and interquartile range and compared using the Mann–Whitney U test. Categorical variables were presented as numbers and percentages and compared using Fisher’s exact test. A value of 2-tailed *p* < 0.05 was considered statistically significant. Statistical analyses were performed using SPSS Statistics 23 (SPSS Inc., Armonk, IL, USA).

The assessment of intra-rater and inter-rater reliability was conducted using the intra-class correlation (ICC) analysis. ICC values falling within the range of 0.41–0.60 were considered moderate, those between 0.61–0.80 were regarded as substantial, and values ranging from 0.81–1.00 were deemed to exhibit an almost perfect level of reliability. 

The independent variable was defined as a baseline CWT, which was measured via abdominal computed tomography. The primary outcome was defined as a 2-year death or heart failure readmission. 

The prognostic impact of CWT was assessed using Cox proportional hazard ratio regression analysis. Adjustments were executed in the multivariable analysis utilizing predetermined variables, notably baseline age, hemoglobin levels, and the logarithm of plasma B-type natriuretic peptides, accounting for their prognostic significance and the number of events. A receiver operating characteristic analysis was performed for the CWT to estimate the primary outcome and calculate a cutoff of CWT to predict the primary outcome. Patients’ cohorts were divided using this cutoff of CWT, and Kaplan–Meier analysis was performed to evaluate the prognostic impact of higher CWT. 

## 3. Results

### 3.1. Baseline Characteristics

A total of 345 patients were included. Median age was 85 (82, 88) years and 99 patients were males (Table 1). All patients had severe aortic stenosis at baseline, with a median aortic valve peak velocity of 4.4 (4.0, 4.9) m/s. No patients had obvious gastrointestinal diseases. The estimated glomerular filtration rate was 48 (37, 61) mL/min/1.73 m^2^, and the plasma B-type natriuretic peptide was 216 (113, 516) pg/mL. The median STS score was 5.2 (3.9, 7.3), and the median EURO II score was 3.4 (2.4, 4.6). 

### 3.2. CWT Measurement

**Figure 3:**
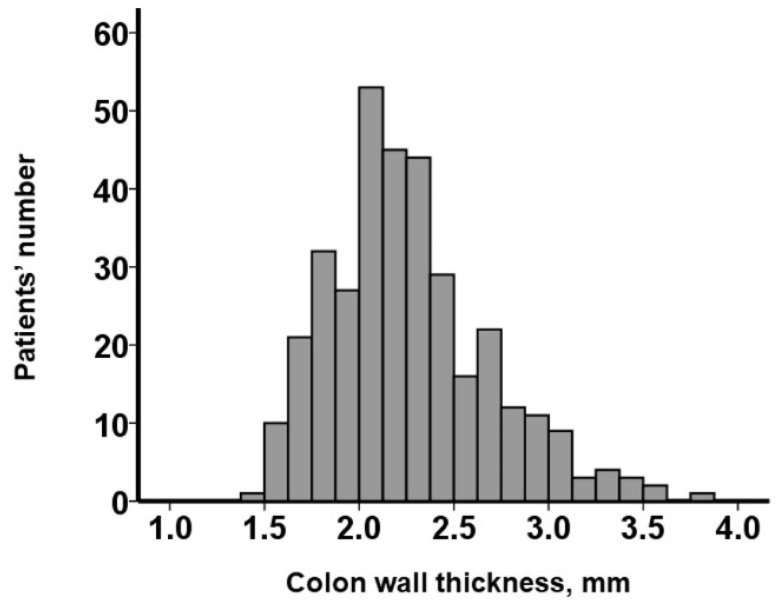
Distribution of baseline colon wall thickness.

CWT levels were distributed widely, with a median value of 2.2 (2.0, 2.5) mm (Figure 3). Regarding the evaluation of intra-observer reliability, the calculated ICC (1, 6) yielded a value of 0.912 (95% confidence interval 0.884–0.934), surpassing the threshold of 0.80, indicating an almost perfect level of reliability (*p* < 0.001). In the assessment of inter-observer reliability, the computed ICC (2, 2) value was 0.874 (95% confidence interval 0.845–0.897). This also exceeded the 0.80 threshold, signifying an almost perfect level of reliability (*p* < 0.001).

Forty-five patients (13%) had a higher CWT above 2.7 mm, which was a calculated cutoff statistically associated with the primary outcome. Patients with moderate or greater mitral regurgitation tended to have higher CWT (*p* = 0.056) (Table 1). Lower pulmonary artery pulsatility index tended to be associated with higher CWT (*p* = 0.087). A higher CWT was significantly associated with more advanced anemia (*p* = 0.012). Patients with higher CWT tended to have lower serum albumin, serum cholinesterase, and total cholesterol, as well as a higher C-reactive protein (*p* < 0.10 for all). Patients with a higher CWT tended to have greater STS scores (*p* = 0.091).

### 3.3. Impact of CWT on the Primary Outcome

Patients were followed up for a median of 719 (365, 730) days after TAVR. During the observation period, 21 patients died, including 2 heart failure causes of death, and 18 patients had heart failure readmissions. As a result, 35 patients die or heart failure readmission (i.e., 4 patients had both heart failure readmission and death). 

CWT level was significantly associated with the primary outcome with a hazard ratio of 2.34 (95% confidence interval 1.14–4.80, *p* = 0.021) and a hazard ratio of 2.02 (95% confidence interval 1.01–4.07, *p* = 0.049) adjusted for pre-specified 3 potential confounders (age, baseline hemoglobin, and baseline logarithm of plasma B-type natriuretic peptide) (Table 2).

CWT tended to be associated with 2-year mortality with a hazard ratio of 2.43 (95% confidence interval 0.98–6.03, *p* = 0.055), whereas it was not significantly associated with a 2-year heart failure readmission with a hazard ratio of 1.42 (95% confidence interval 0.49–4.16, *p* = 0.52).

### 3.4. Risk Stratification Using a Cutoff of CWT

**Figure 4:**
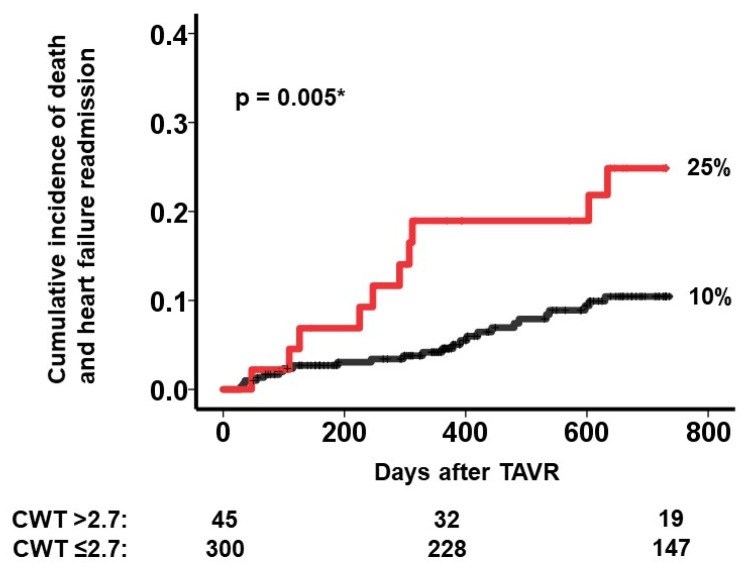
The cumulative incidence of the primary outcome during the 2-year observation period after TAVR stratified by the cutoff of colon wall thickness. Patients were stratified by the cutoff of colon wall thickness. The primary outcome was defined as death or heart failure readmission. * *p* < 0.05 by log-rank test.

A cutoff of CWT to predict the primary outcome was calculated as 2.7 mm with a sensitivity of 0.29, a specificity of 0.89, and an area under the curve of 0.61. A CWT >2.7 mm was significantly associated with the primary outcome with a hazard ratio of 2.72 (95% confidence interval 1.31–5.66, *p* = 0.008). Patients with CWT >2.7 mm had a significantly higher 2-year cumulative incidence of the primary outcome than others (25% versus 10%, *p* = 0.005; Figure 4).

We also attempted to use a median value of CWT 2.2 mm to the risk stratification patient cohort. A CWT >2.2 mm was not significantly associated with the primary outcome with a hazard ratio of 1.47 (95% confidence interval 0.75–2.90, *p* = 0.26). 

## 4. Discussion

Within this study, we conducted an assessment of the prognostic significance of intestinal dysfunction, as shown by CWT, indicating intestinal wall edema among patients diagnosed with severe aortic stenosis undergoing TAVR. Our findings suggest a tendency for CWT to correlate with compromised right ventricular function, as denoted by a decreased pulmonary artery pulsatility index score. Additionally, elevated CWT demonstrated a propensity to align with higher levels of C-reactive protein and indications of malnutrition. Notably, CWT emerged as an independent predictor for the occurrence of death and readmission due to heart failure within a two-year period following TAVR. 

### 4.1. Heart Failure and Intestinal Dysfunction

Although detailed mechanisms remain uncertain, an intestinal–cardiovascular relationship has been receiving great concern so far to better understand the physiological mechanism of heart failure as a systemic disease involving multiple organs [7,8]. The presence of heart failure may reduce intestinal blood flow and cause intestinal congestion, both of which trigger intestinal dysfunction [10,11]. Intestinal dysfunction generally accompanies intestinal wall edema, which can be assessed via CWT [14]. The median value of CWT in our cohort was higher than those of the previously reported healthy cohort [16], probably indicating systemic congestion and impaired systemic circulation due to the presence of severe aortic stenosis [17].

A lower pulmonary artery pulsatility index, which was a recently proposed variable in order to assess right ventricular function, tended to be associated with higher CWT. Other hemodynamics, including E/e’ ratio, were not associated with CWT. This is probably because the hemodynamics of our patients were relatively well controlled before TAVR.

Intestinal dysfunction causes dysbiosis, which triggers systemic inflammation via intestinal barrier dysfunction and bacterial translocation [12]. C-reactive protein also tended to be associated with CWT in our study. Such an activated systemic inflammation further progresses heart failure and vice versa. 

### 4.2. Intestinal Dysfunction and Cardiac Cachexia

Intestinal dysfunction impairs the absorption of nutrients and progresses sarcopenia [9]. Induced systemic inflammation facilitates lymphocyte apoptosis via activated cytokine families and further progresses to malnutrition [18]. Lymphatic stasis in the thoracic duct due to central venous congestion also leads to the loss of lymphocytes in the intestine [18]. Our patients with a higher CWT consistently had lower cholinesterase and hypoalbuminemia.

Recent studies focus on comorbidity as a prognostic risk factor for TAVR candidates rather than heart failure-related parameters, given that most TAVR candidates are elderly patients with multiple comorbidities that cannot be treated directly via TAVR alone [6]. Thus, it should not be surprising that a higher CWT, which may be associated with intestinal dysfunction and malnutrition, was associated with worse clinical outcomes after TAVR. The prognostic impact of CWT on heart failure readmission did not reach significant levels. Most of the causes of death were not heart failure. Similar to other extra-cardiac risk factors, CWT may have a negative prognostic impact on non-cardiovascular death after TAVR, even though TAVR can ameliorate the stenotic aortic valve and unload the left ventricle.

### 4.3. Clinical Implication of Our Findings

CWT can be measured easily without any expert techniques or equipment. Computed tomography imaging is routinely obtained before TAVR to discuss its indication and applicability. We do not refute the indication of TAVR for individuals exhibiting elevated CWT. Instead, our discoveries hold utility in facilitating shared decision making among clinicians, patients, and their families, providing insights into the anticipated clinical outcomes subsequent to TAVR. 

Patients exhibiting elevated CWT levels post-TAVR warrant meticulous monitoring. Addressing congestion through the judicious use of suitable diuretics could potentially disrupt the detrimental cycle associated with intestinal–cardiovascular syndrome by mitigating intestinal edema [19]. Notably, a prior study delineated a U-shaped relationship between CWT and defecation frequency [14]. Implementing appropriate interventions to manage dyschezia, such as adjusting laxative usage and/or dietary intake, may foster stabilization of the intestinal microbiota, consequently interrupting the progression of the intestinal–cardiovascular syndrome [20].

### 4.4. Limitations

This investigation encompassed a cohort of moderate scale sourced from a solitary center. Due to the limited occurrence of events, the array of potential confounding variables encompassed in the multivariable analysis was restricted. Moreover, unidentified confounding factors might have been present but were not evaluated. We referenced the previous literature to measure CWT, but the methodology used to measure CWT has not yet been robustly established. The intestinal–cardiovascular relationship is complex. We aimed to evaluate the prognostic impact of CWT and did not perform detailed analyses to clarify its mechanism. For example, we did not collect data on the defecation number and the gut microbiome. Also, detailed causality between CWT and clinical outcomes remains uncertain. We measured CWT only one time before TAVR, and its trajectory after TAVR remains unknown.

## 5. Conclusions

CWT, serving as an indicator of intestinal wall edema resultant from intestinal dysfunction, exhibited an association with mid-term mortality and heart failure readmission in elderly patients diagnosed with severe aortic stenosis undergoing TAVR. Further exploration into the intricate causative relationship between these factors and the clinical significance of implementing assertive interventions to ameliorate CWT stands as a critical area for future investigation.

## Figures and Tables

**Table 1:** Baseline characteristics.

	Total (*N* = 345)	CWT > 2.7 mm (*N* = 45)	CWT ≤ 2.7 mm (*N* = 300)	*p*-c
Demographics				
Age, years	85 (82, 88)	87 (82, 90)	85 (82, 88)	0.23
Male sex	99 (29%)	14 (31%)	85 (28%)	0.41
Body surface area, m^2^	1.38 (1.30, 1.52)	1.35 (1.27, 1.44)	1.39 (1.31, 1.52)	0.79
Systolic blood pressure, mm Hg	116 (106, 127)	118 (106, 124)	115 (106, 127)	0.94
Pulse rate, bpm	71 (63, 78)	74 (71, 78)	70 (63, 78)	0.52
Comorbidity				
Hypertension	252 (73%)	35 (78%)	217 (72%)	0.31
Dyslipidemia	165 (48%)	19 (42%)	146 (49%)	0.25
Diabetes mellitus	61 (18%)	8 (18%)	53 (18%)	0.60
Atrial fibrillation	56 (16%)	4 (9%)	52 (17%)	0.11
History of stroke	45 (13%)	3 (7%)	42 (14%)	0.12
Coronary heart disease	88 (26%)	14 (31%)	74 (25%)	0.23
Laboratory data				
Hemoglobin, g/dL	11.4 (10.0, 12.4)	10.3 (8.9, 11.7)	11.4 (10.1, 12.5)	0.012 *
Serum albumin, g/dL	3.8 (3.5, 4.0)	3.6 (3.3, 4.0)	3.8 (3.5, 4.1)	0.068
Serum cholinesterase, U/L	234 (195, 278)	205 (169, 281)	238 (198, 277)	0.063
Serum sodium, mEq/L	141 (139, 142)	140 (137, 142)	141 (139, 142)	0.074
Serum potassium, mEq/L	4.4 (4.1, 4.6)	4.4 (4.1, 4.7)	4.3 (4.0, 4.6)	0.94
Serum total bilirubin, mg/dL	0.5 (0.4, 0.7)	0.5 (0.4, 0.6)	0.5 (0.4, 0.7)	0.76
eGFR, mL/min/1.73 m^2^	48 (37, 61)	41 (31, 58)	49 (38, 64)	0.24
Plasma B-type natriuretic peptide, pg/mL	216 (113, 516)	268 (151, 563)	200 (109, 449)	0.30
Total cholesterol, mg/dL	165 (147, 194)	156 (142, 192)	167 (148, 194)	0.060
C-reactive protein, mg/dL	0.11 (0.04, 0.36)	0.14 (0.04, 0.83)	0.11 (0.04, 0.33)	0.087
Echocardiography				
Left ventricular end-diastolic diameter, mm	46 (42, 51)	46 (40, 51)	46 (42, 51)	0.58
Left ventricular ejection fraction, %	64 (54, 70)	67 (55, 72)	63 (54, 70)	0.62
Left atrial diameter, mm	43 (39, 49)	45 (41, 50)	43 (38, 49)	0.28
Moderate or greater MR	34 (10%)	8 (18%)	26 (9%)	0.056
Moderate or greater TR	18 (5%)	3 (7%)	15 (5%)	0.64
E/e’ ratio	16.5 (12.6, 22.7)	17.3 (14.0, 23.3)	16.5 (12.3, 22.6)	0.25
Aortic valve peak velocity, m/s	4.4 (4.0, 4.9)	4.5 (4.0, 4.9)	4.4 (4.0, 4.8)	0.46
Mean aortic valve velocity, m/s	46 (38, 57)	47 (38, 57)	46 (38, 56)	0.55
Aortic valve area, cm^2^	0.54 (0.44, 0.67)	0.54 (0.47, 0.71)	0.54 (0.44, 0.66)	0.58
Hemodynamics				
CVP, mm Hg	5 (3, 7)	5 (3, 8)	5 (3, 7)	0.72
Mean pulmonary artery pressure, mm Hg	19 (16, 23)	19 (16, 25)	19 (15, 23)	0.92
PAWP, mm Hg	11 (8, 15)	11 (7, 16)	11 (8, 15)	0.81
Cardiac index, L/min/m^2^	2.7 (2.3, 3.0)	2.6 (2.3, 2.9)	2.7 (2.3, 3.0)	0.95
CVP/PCAWP ratio	0.45 (0.33, 0.57)	0.44 (0.30, 0.57)	0.45 (0.33, 0.57)	0.98
PAPi	3.5 (2.4, 6.0)	3.3 (2.4, 4.8)	3.6 (2.6, 6.0)	0.087
RVSWI, g/m	7.3 (5.3, 9.9)	7.0 (5.4, 9.6)	7.4 (5.1, 9.8)	0.32
Scores				
STS score	5.2 (3.9, 7.3)	5.6 (4.5, 9.3)	5.1 (3.8, 7.2)	0.091
EURO II score	3.4 (2.4, 4.6)	3.6 (2.9, 4.6)	3.4 (2.4, 4.5)	0.24
GNRI	97 (91, 104)	96 (86, 105)	98 (91, 105)	0.45
Medication				
Beta-blocker	112 (32%)	15 (33%)	97 (32%)	0.51
Renin-angiotensin system inhibitor	213 (62%)	26 (58%)	187 (62%)	0.33
Mineralocorticoid receptor antagonist	98 (28%)	15 (33%)	83 (28%)	0.27
Loop diuretics	189 (55%)	25 (56%)	164 (55%)	0.52

CWT, colon wall thickness; eGFR, estimated glomerular filtration rate; MR, mitral regurgitation; TR, tricuspid regurgitation; CVP, central vein pressure; PAWP, pulmonary artery wedge pressure; PAPi, pulmonary artery pulsatility index; RVSWI, right ventricular stroke work index; STS, society of thoracic surgeons; GNRI, geriatric nutritional risk index. Continuous variables were stated as median (25% interquartile, 75% interquartile) and compared between the two groups using the Mann–Whitney U test. Categorical variables were stated as numbers (percentages) and compared between the two groups using the Fischer’s exact test. * *p* < 0.05.

**Table 2:** Prognostic impact of colon wall thickness on the primary outcome.

	Hazard Ratio (95% Confidence Interval)	*p*-Value
Colon wall thickness, cm	2.02 (1.01–4.07)	0.049 *
Hemoglobin, g/dL	0.81 (0.65–1.02)	0.075
Age, years	1.03 (0.96–1.10)	0.47
Logarithm of plasma BNP, pg/mL	1.48 (0.69–3.17)	0.32

BNP, B-type natriuretic peptide. The cox proportional hazard ratio regression analysis was performed to evaluate the prognostic impact of colon wall thickness and pre-specified 3 potential confounders. * *p* < 0.05.

## Data Availability

Data are available upon reasonable reason by the corresponding author.
